# Recent progress on deep eutectic solvents in biocatalysis

**DOI:** 10.1186/s40643-017-0165-5

**Published:** 2017-07-21

**Authors:** Pei Xu, Gao-Wei Zheng, Min-Hua Zong, Ning Li, Wen-Yong Lou

**Affiliations:** 1 Laboratory of Applied Biocatalysis, School of Food Sciences and Engineering, South China University of Technology, Guangzhou, 510640 China; 2 State Key Laboratory of Pulp and Paper Engineering, South China University of Technology, Guangzhou, 510640 China; 3 State Key Laboratory of Bioreactor Engineering, East China University of Science and Technology, Shanghai, 200237 China

**Keywords:** Deep eutectic solvents, Biocatalysis, Catalysts, Biodegradability, Influence

## Abstract

Deep eutectic solvents (DESs) are eutectic mixtures of salts and hydrogen bond donors with melting points low enough to be used as solvents. DESs have proved to be a good alternative to traditional organic solvents and ionic liquids (ILs) in many biocatalytic processes. Apart from the benign characteristics similar to those of ILs (e.g., low volatility, low inflammability and low melting point), DESs have their unique merits of easy preparation and low cost owing to their renewable and available raw materials. To better apply such solvents in green and sustainable chemistry, this review firstly describes some basic properties, mainly the toxicity and biodegradability of DESs. Secondly, it presents several valuable applications of DES as solvent/co-solvent in biocatalytic reactions, such as lipase-catalyzed transesterification and ester hydrolysis reactions. The roles, serving as extractive reagent for an enzymatic product and pretreatment solvent of enzymatic biomass hydrolysis, are also discussed. Further understanding how DESs affect biocatalytic reaction will facilitate the design of novel solvents and contribute to the discovery of new reactions in these solvents.

## Introduction

Biocatalysis, defined as reactions catalyzed by biocatalysts such as isolated enzymes and whole cells, has experienced significant progress in the fields of either biocatalysts or reaction medium during the past decades. One noteworthy example is the greener synthetic pathway developed by Savile for the redesigned synthesis of sitagliptin, an active ingredient used to treat diabetes (Savile et al. 2010). Generally, biocatalysis refrains many weaknesses, such as the lack of enantio-, chemo- and regioselectivity and utilization of metal catalysts in organic synthesis in the production of valuable compounds by the chemical method (Bommarius and Paye 2013).

Along with the rapid emergence of new biocatalyst technology, the shift to green chemistry puts high stress on the biocatalytic process. Among the Twelve Principles of Green Chemistry developed by Anastas and Warner (1998), one concept is “Safer Solvents and Auxiliaries”. Solvents represent a permanent challenge for green and sustainable chemistry due to their vast majority of mass used in catalytic processes (Anastas and Eghbali 2010). Environmental benignity is the ideal pursuit of exploiting each generation of solvents.

Solvents for a biocatalysis reaction have experienced several generations of development. Water has been considered as the greenest solvent considering its quality and quantity. However, the high polarity of the water molecule hinders its application in all biocatalytic reactions, because of some substrates’ insolubility in aqueous solution due to their high hydrophobicity. Traditional organic solvents (water miscible or water immiscible), in the form of co-solvents or second phase, can provide solutions for the above-described challenges to some degree. But inevitably, organic solvents face their own disadvantages such as high volatility, inflammability and activity inhibition to the biocatalyst. Ionic liquids (ILs) are the first enzyme-compatible untraditional media developed by the green and sustainable concept (given their low vapor pressure). Numerous reactions, e.g., hydrolytic and redox reactions as well as formation of C–C bond, have been successfully performed in such ILs-containing media (Potdar et al. 2015; Roosen et al. 2008; van Rantwijk and Sheldon 2007). Despite the excellent performance of ILs in biocatalysis, more doubts about their ungreenness and environmental influence have been gradually presented (Zhao et al. 2007; Thuy Pham et al. 2010; Petkovic et al. 2012).

**Fig. 1:**
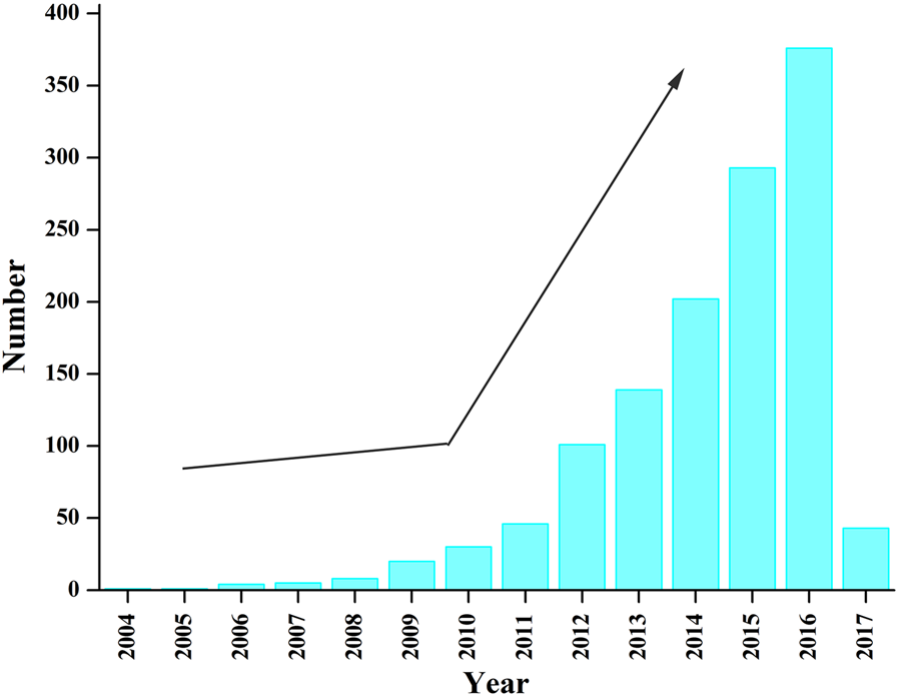
The number of published papers on DESs starting from 2004 (adapted from ISI Web of Knowledge)

Deep eutectic solvents (DESs), the recognized alternative of ILs, first came to the public vision in 2001 (Abbott et al. 2001). Since then, research on DESs faced a prosperous increase in many fields, such as extraction, materials synthesis, and biotransformation (Atilhan and Aparicio 2016; Carriazo et al. 2012; Dai et al. 2013b; Garcia et al. 2015; Gonzalez-Martinez et al. 2016; van Osch et al. 2015). A brief statistics of the published literature starting form 2004 is presented in Fig. 1. This review will provide an overview of the (un)greenness of DESs and recent applications in biocatalysis. Finally, how DESs affect biocatalytic reactions are discussed.

## DESs and their properties

### DESs

DESs are eutectic mixtures of salts and hydrogen bond donors (HBDs) with melting point low enough to be used as solvents. A eutectic liquid is generally formed by two solids at eutectic temperature, in which the process follows a thermal equilibrium. A deep eutectic system refers to a eutectic mixture with a melting point much lower than either of the individual components. A classic example is that ChCl/urea (1:2 mol ratio) mixture has a melting point of 12 °C (far lower than the melting point of the ChCl and urea, 302 and 133 °C, respectively), which makes it a liquid to be used as a solvent at room temperature. In addition, the complex hydrogen bonding network existing in DES also makes it a special solvent for different applications (Hammond et al. 2016).

**Fig. 2:**
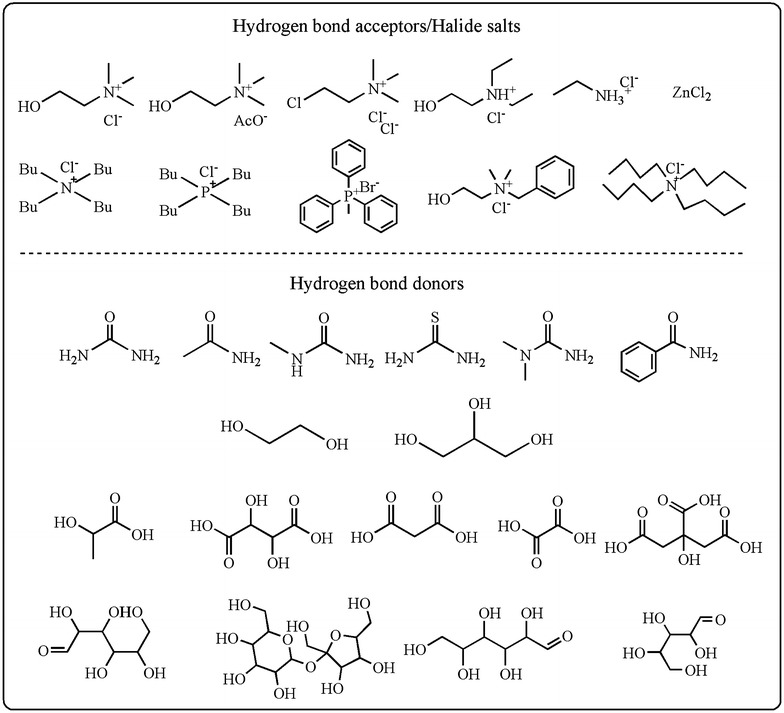
Examples of chemicals for DESs synthesis

Up to now, many kinds of DESs have been developed with various compounds (Fig. 2). They are generally prepared by mixing a hydrogen bond acceptor (HBA), such as quaternary ammonium salt with a metal salt or HBD at moderate temperature with constant stirring. The first examples of DESs were prepared by heating ZnCl_2_ with a series of quaternary ammonium salts, in which choline chloride is the HBA obtained at the surprisingly lowest melting point of 23–25 °C (Abbott et al. 2001). In 2014, Abbott et al. proposed a general formula of Cat^+^X^−^ *z*Y for DESs, where Cat^+^ refers to ammonium, phosphonium or sulfonium cation and X generally represents a halide anion (Smith et al. 2014). This formula represents four classes of DESs according to the nature of the components used.

Class I: Cat^+^X^−^ *z*MCl*x*, M = Zn, Sn, Fe, Al, Ga, In.
Class II: Cat^+^X^−^ *z*MCl*x* *y*H_2_O, M = Cr, Co, Cu, Ni, Fe.
Class III: Cat^+^X^−^ *z*RZ, Z = CONH_2_, COOH, OH.
Class IV: MCl*x* + RZ = MCl_*x*-*1*_^+^·RZ + MCl_*x*+*1*_^−^, M = Al, Zn and Z = CONH_2_, OH.

The main difference between Class II DESs and I is the inherent water, which makes it possible for many hydrated metal halide salts to be used as HBDs to synthesize DESs, since they have relatively lower cost.

Another set of DESs, “natural deep eutectic solvent (NADES)”, are developed by utilizing many primary metabolites, such as amino acids, sugars, choline and organic acid (Choi et al. 2011). NMR-based metabolomics analysis indicated that NADES indeed existed as the third liquid other than water and lipid in an organism. For example, a mixture of sucrose, fructose and glucose (1:1:1, molar ratio) forms a clear and uniform liquid at room temperature. They can help plants survive anhydrobiosis and play a role in the synthesis of intracellular macromolecules. These NADES may preserve the linkage between understanding of cellular metabolism and physiology.

### Properties

DESs have some special physical traits which are very useful for biocatalytic process, i.e., a wide liquid state range, nonflammability and low volatility (Abbott et al. 2011; Paiva et al. 2014; Smith et al. 2014; Zhang et al. 2012). Low temperature of freezing point (T*f*) makes it possible for DESs to remain as a liquid in a biocatalytic process, because most biocatalytic reactions proceed in a liquid environment, in either monophasic or biphasic system. As reported, the *T*_*f*_ of a DES depends on many factors. Increased hydroxyl group of HBD increased the value of T_*f*_. For example, the HBD in ChCl/Gly has three hydroxyl groups and melts at −40 °C, while the HBD in ChCl/EG has two hydroxyl groups and melts at −66 °C (Zhang et al. 2012). DESs with different mole ratios of HBA to HBD also show different T_*f*_, but no obvious tendency was observed. The glycerol-based DESs with methyl triphenyl phosphonium bromide as HBA (1:2–1:4, molar ratio) have a T_*f*_ range from −5.5 to 15.8 °C (Shahbaz et al. 2011). Besides, DESs are commonly not flammable and have very low volatility, because their individual components are generally solids or inflammable liquid. These two properties decrease the risk of explosion and burning for an organic reaction if DESs are used as solvents instead of volatile organic solvents, thus making it safe to operate. What is more, density and viscosity are the other two important characteristics which affect the mass transfer when selecting a solvent for use. Generally, the viscosity of DESs lies in the constitution and is higher than that of the molecular solvent ethanol (Smith et al. 2014). The specific relationship between these factors has been documented at length in another review (Zhang et al. 2012). Most properties change as a function of temperature (Zhao et al. 2015). For instance, as the temperature increases, the density and viscosity decrease.

Two other advantages of DESs over ILs are easier preparation and lower cost. Typical preparation of DESs involves a mixing of the components in a certain molar ratio (e.g., 1:2 of HBA/HBD) with constant stirring at moderate temperature. This simple synthesis is 100% atom economic, requiring no further purification. Besides, the commonly used components of DESs, such as ChCl and glycerol, are all from natural materials. Especially, NADESs are absolutely formed by primary metabolites, such as glucose, malic acid and succinic acids (Dai et al. 2013a). These raw materials exist naturally and have relatively low cost of production. By contrast, the common preparation of ILs has two steps: first alkylation of an amine/phosphine/sulfide to afford an intermediate salt, followed by anion exchange to give the ILs (Hallett and Welton 2011). The complexity of synthesis will spontaneously increase the production cost. Thus, the synthesis of DESs is easier and costs less than that of ILs.

### Toxicity

A good question about whether DESs are toxic was proposed in 2013 (Hayyan et al. 2013b). The answer for that is some of them are toxic. Most of them are basically soluble in water. Especially in the cases of Class I and Class II DESs, the metal ion will inevitably leave a trace in the environment, thus exerting pressure on recycling these solvents.

To date, only a few groups have investigated the toxicological properties of DESs in detail (Table 1). Hayyan and co-workers first investigated the toxicity and cytotoxicity of ChCl- and phosphonium-based DESs, of which glycerine, ethylene glycol, triethylene glycol and urea were used as the HBDs (Hayyan et al. 2013a, b). They pointed out that the toxicity and cytotoxicity of different DESs varied with the structure of the components used. ChCl-based DESs were less toxic than phosphonium-based DESs. Afterward, the toxicity of ammonium-based DESs was evaluated in in vitro cell lines and in vivo animal models (Hayyan et al. 2015). In this study, the DESs tested showed higher toxicity than their individual components,which was consistent with other DESs reported previously. Later, some ternary DESs consisting of ChCl, water and natural substance were also examined for their cytotoxicity (Hayyan et al. 2016). Selection of DESs parents is very important for the toxicity of resulting DESs. The IC_50_ of ChCl/Gly/H_2_O toward Hela S3 cell was almost 2.4-fold higher than that of ChCl/fructose/H_2_O (427 mM vs 177 mM), though in the former DES Gly had higher concentration (1:2:1 vs 5:2:5, respectively). Glycerol is a wildly used sweetener and humectant in the food industry and used in strain preservation; so the toxicity of glycerol-based DESs is expectable to some degree.

**Table 1:** The toxicity of some DESs

DESs	Organism	Toxicity comments	References
MTPB/Gly (1:3) MTPB/EG (1:3) MTPB/TEG (1:3)	*Escherichia coli, Staphylococcus aureus, Pseudomonas aeruginosa, Bacillus subtilis, Artemia salina*	All the DESs showed toxic effect on some bacteria The cytotoxicity of DESs was much higher than their individual components	Hayyan et al. (2013a)
ChCl/Gly (1:3) ChCl/EG (1:3) ChCl/U (1:3) ChCl/TEG (1:3)	*Escherichia coli, Staphlococcus aureus, Pseudomonas aeruginosa, Bacillus subtilis, Artemia salina*	All the DESs showed no toxic effect on the bacteria The cytotoxicity of DESs was much higher than their individual components	Hayyan et al. (2013b)
ChCl/Gly (1:3) ChCl/EG (1:3) ChCl/TEG (1:3) ChCl/U (1:3)	PC3, A375, HepG2, HT29, MCF-7, OKF6, H413, ICR mice	The cytotoxicity of DESs varied from various cell lines. The toxic effects of DESs were higher than their individual components	Hayyan et al. (2015)
ChCl or ChOAc/U (1:1) ChCl or ChOAc/Gly (1:1) ChCl or ChOAc/A (1:1) ChCl or ChOAc/EG (1:1)	*Escherichia coli*	0.75 M DES could afford an inhibition index of 72.8–93.8% for the bacterium and was more toxic then the components	Wen et al. (2015)
ChCl/EG (1:2) ChCl/Gly (1:2) ChCl/U (1:2) DAC/EG (1:2) EAC/Gly (1:2) DAC/MA (1:1) DAC/ZnN (1:1) DAC/ZnCl_2_ (1:2)	*Aspergillus niger, Cyprinus carpio* fish	Metal salt-containing DESs were most toxic than others. DAC-based DESs were less toxic than ChCl-based DESs	Juneidi et al. (2015)
20 kinds of NADESs	*Staphylococcus aureus, Listeria monocytogenes, Escherichia coli, Salmonella enteritidis*	All the DESs except for acid-containing DESs showed no toxic effect on the bacteria	Zhao et al. (2015)
ChCl/ZnCl_2_ (1:2) ChCl/U (1:2) ChCl/Gly (1:3) ChCl/EG (1:3) ChCl/DEG (1:2) ChCl/TEG (1:3) ChCl/Fru (2:1) ChCl/Glc (2:1) ChCl/PTSA (1:3) ChCl/MA (1:1)	*Phanerochaete chrysosporium, Aspergillus niger, Lentinus tigrinus, Candida cylindracea*	ZnCl_2_, PTSA and MA DESs had the most toxic effect	Juneidi et al. (2016)
ChCl/Fru/water (5:2:5) ChCl/Glc/water (5:2:5) ChCl/Suc/water (4:1:4) ChCl/Gly/water (1:2:1) ChCl/malonic acid (1:1)	HelaS3, CaOV3, B16F10, MCF-7	NADESs except malonic acid as HBD are less toxic than DESs	Hayyan et al. (2016)
Choline and geranate (1:2)	*M. tuberculosis, S. aureus, P. aeruginosa* et al.	The DES is so toxic that it can act as a broad-spectrum antiseptic agent	Zakrewsky et al. (2016)
ChCl/Fru (2:1) ChCl/Glc (2:1) DAC/TEG (1:3)	HelaS3, PC3, AGS, A375, MCF-7, WRL-68	The DESs (ChCl/Fru and ChCl/Glc, 98 mM ≤ EC_50_ ≤ 516 mM) were less toxic than DAC/TEG(34 mM ≤ EC_50_ ≤ 120 mM)	Mbous et al. (2017)

However, the toxic DESs can be used for beneficial purposes, e.g., as effective anti-bacterial agents. The DES choline/geranate can inhibit the growth of some bacteria, fungi and viruses, making it a possible broad-spectrum antimicrobial agent (Zakrewsky et al. 2016).

In our work, a method of bacterial growth inhibition was used to assess the toxicology of four types of DESs based on ChCl, which was coupled with amine-, alcohol-, sugar- and organic acid-based HBDs (Zhao et al. 2015). The organic acid-based DESs significantly inhibited bacterial growth, suggesting the limitation of utilizing organic acid when designing DESs. This finding was in accordance with Hayyan’s study, which found that ChCl/MA was greatly toxic toward the tested mammalian cells (Hayyan et al. 2016). The other three types of DESs displayed less toxicity to the tested Gram-positive (*Staphylococcus aureus* and *Listeria monocytogenes*) and Gram-negative (*Escherichia coli* and *Salmonella enteritidis*) bacteria. Interestingly, a further MIC test demonstrated that the two Gram-negative bacteria were less sensitive than Gram-positive bacteria toward the organic acid-based DESs. A mechanism of this difference may be related to the oligosaccharide constituent.

All the examples give us information, that is, reasonable design and post-processing of DESs are very important for us to utilize this solvent. Novel DESs can be made according to the properties of parent components for special use. Some DESs showed toxicity to some extent, but there is limitation for these studies, due to the fact that the tested organisms are different with what we use in a biocatalytic process. For a typical reaction, one can determine the effect of DESs they use on the biocatalyst, under the guidance of previous researches.

### Biodegradability

Biodegradability is another important property except toxicity when talking about the “greenness” of DESs. A large proportion of DESs are considered as “readily biodegradable”, possibly because most of the components forming DESs are natural products (Radosevic et al. 2015; Zakrewsky et al. 2016). Taking the NADES, for instance, all the individual components come from natural materials and can be metabolized by different kinds of organisms in nature. Glycerol can enter the pathway of glycolysis or gluconeogenesis for final metabolism and the sugar portion can also be directly utilized (Mbous et al. 2017).

Table 2 shows the recent studies which reported the biodegradability of DESs. In Radosevic’s work, the closed bottle test (OECD) was used according to the OECD guideline 301 D (1992D), and all the tested ChCl-based DESs (ChCl:Gly, ChCl:Glc, and ChCl:OA) provided a biodegradation level of over 60% after 14 days (Radosevic et al. 2015). The highest biodegradability level was observed up to 96% with ChCl:Gly. These findings for DES biodegradability were in accordance with our study, in which all the ChCl-based DESs had degradability of over 69.3% after 28 days (Zhao et al. 2015). However, Wen et al. found a different conclusion (Wen et al. 2015). They found that only two (ChCl/U and ChCl/A) of the eight DESs tested could be regarded as readily biodegradable. The two DESs showed a degradability close to 80%; nevertheless, the others provided a degradability below 50% in contrast. In the same year, Juneidi et al. also presented a comprehensive study on the biodegradability for cholinium-based DESs (Juneidi et al. 2015). It is worth mentioning that this work involved in the toxicity and biodegradability of DESs-containing a metal salt and a hydrated metal salt (i.e., ZnCl_2_ and ZnN). There was a significant difference between the degradability of DAC:ZnN and DAC:ZnCl (about 80% vs 62%). Even the biodegradability of the former was better than that of the conventional IL [BMPyr][NTf_2_] (77%). The inherent structure of HBAs and HBDs is the basic factor which determines the biodegradability of different DESs, i.e., the number of hydroxy groups (Radosevic et al. 2015). For a special DES, the difference in result between various reports should be attributed to the difference of the measure condition, the molar ratio of DESs and the wastewater microorganisms.

**Table 2:** The biodegradability of some DESs

DESs	Assay method	Resource of microorganism	DES concentration (mg/L)	Comments	Reference
ChCl/Glc (2:1) ChCl/OA (1:1) ChCl/Gly (1:2)	Closed bottle test	Effluent from an urban wastewater treatment plant	100	Over 60% of biodegradation level after 14 days	Radosevic et al. (2015)
20 kinds of NADESs	Closed bottle test	Fresh lake water	3	All DESs had a biodegradation level over 69.3% after 28 days. The acid-based DESs were degraded slower than others	Zhao et al. (2015)
ChCl or ChOAc/U (1:1) ChCl or ChOAc/Gly (1:1) ChCl or ChOAc/A (1:1) ChCl or ChOAc/EG (1:1)	Closed bottle test	Activated sludge from wastewater treatment plant	4	Only ChCl/U and ChCl/A were readily biodegradable	Wen et al. (2015)
ChCl/EG (1:2) ChCl/Gly (1:2) ChCl/U (1:2) DAC/EG (1:2) EAC/Gly (1:2) DAC/MA (1:1) DAC/ZnN (1:1) DAC/ZnCl_2_ (1:2)	Closed bottle test	Wastewater from secondary effluent treatment plant	5	All DESs were referred to as readily biodegradable. ChCl-based DESs had higher biodegradability than DAC-based DESs	Juneidi et al. (2015)

In summary, DESs exhibit relatively low toxicity toward organisms in a laboratory scale and could be classified as biodegradable green solvents. These benign characteristics may attract more attention of researchers to shift from the classical imidazolium and pyridinium ILs to DESs. More organic chemistry reactions could be explored in such solvents.

## The application of DESs in biocatalysis

Recent decades have witnessed the dramatic publications of ILs in chemical synthesis with isolated enzymes or whole cells (Wang et al. 2012). By comparison, only dozens of studies about the biocatalysis in DESs have been published. The following parts will introduce the recent applications of DESs in biocatalytic reactions, in which DESs played different roles such as solvent, co-solvent or substrate.

### DESs as solvent for enzyme catalysis

As a new generation of solvent, DESs have distinctive characteristics, e.g., abundant hydrogen bond and low T_*f*_. The components of DESs may display inhibitory effect on enzyme activity. For example, urea could induce the unfolding of enzymes and further make them inactive (Attri et al. 2011). Even so, some hydrolases still exhibited better activity in DES compared with ILs and catalyzed a lot of hydrolysis and transesterification reactions as well as Henry reaction and aldol reaction in this special solvent.

#### Lipase

**Fig. 3:**
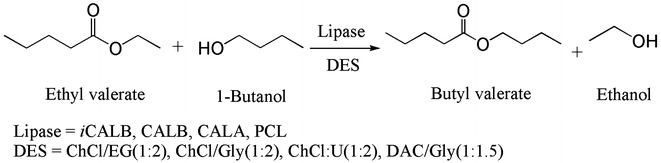
Lipase-catalyzed transesterification of ethyl valerate to butyl valerate in DESs (adapted from Gorke et al. 2008)

Lipases, which are commonly investigated in an organic environment, are the popular choices for enzymatic reaction carried out in DESs (Table 3). In 2008, Kazlauskas et al. first tried the lipase-catalyzed biotransformation reaction in DESs (Fig. 3) (Gorke et al. 2008). Four lipases (iCALB, CALB, CALA and PCL) were investigated for their transesterification activity in eight DES-containing systems. In glycerol-containing DESs, all the enzymes showed conversions of ethyl valerate to varying degrees. Especially in ChCl/Gly, PCL exhibited the lowest conversion (22%), still much higher than that in toluene (5.0%). Interestingly, the alcohol component, either ethylene glycol or glycerol, could compete with 1-butanol. When the concentration of the substrate 1-butanol was much lower than the EG component (400 mM vs 10 M), the CALB-catalyzed reaction still displayed a nearly equal amount of product esters. The reason for this may be the high hydrogen bond network, which hinders the reaction of the alcohol components with the substrate. Additionally, the iCALB exhibited great aminolysis activity in ChCl:Gly-containing system (52 μmol h^−1^ mg^−1^), which was five times higher than in BMIM[BF_4_] (9 μmol h^−1^ mg^−1^). This example opened the possibilities for exploring various biocatalytic reactions in DES-containing solvents.

**Table 3:** Examples of lipase-catalyzed reactions in DESs

Enzyme	DES	Substrate	Product	Comments	Reference
iCALB, CALB, CALA and PCL	ChCl/Gly (1:2) ChCl/U (1:2) EAC/Gly (1:1.5)	Ethyl valerate with 1-butano	Butyl valerate	ChCl/Gly showed good compatibility with all the lipases	Gorke et al. (2008)
iCALB	ChCl/U (1:2) ChOAc/Gly	Miglyol oil 812	Triglyceride	High yield showed the potential of DES as solvent in the biodiesel synthesis	Zhao et al. (2011a)
iCALB	ChCl/Gly (1:2) ChCl/U (1:2)	Vinyl ester and alcohols	Esters	Some HBDs could compete with the substrate	Durand et al. (2012)
Novozyme 435	ChCl/Gly (1:2)	Soybean oil	Biodiesel	This work expanded the substrate spectrum of biodiesel synthesis	Zhao et al. (2013)
Novozyme 435	ChCl/Gly (1:2) ChCl/U (1:2)	Phenolic esters	Phenolic esters	Water content in DES–water mixtures makes great difference on reaction efficiency	Durand et al. (2013)
iCALB	ChCl/U	Phenolic esters	Phenolic esters	First investigated the effect of water activity and U content on product yields	Durand et al. (2014)
Lipozyme CalB L Novozym 435	ChCl/U (1:2) ChCl/GlyZ (1:1)	Oleic acid and decanol	Decyl oleate	Esters product could be easily separated from the aqueous reaction mixtures	Kleiner and Schörken (2015)
Novozyme 435	ChCl/U (1:2) ChCl/Glc	Glucose and vinyl hexanoate	Glucose-6-*O*-hexanoate	Glucose component in DESs can act as substrate	Pöhnlein et al. (2015)
Lipozyme TLIM, Novozym 435	ChAc/U (2:1)	Glucose with fatty acid vinyl esters; methyl glucoside with fatty acids	Sugar fatty acid esters	Utilization of combination of ILs and DESs	Zhao et al. (2016)
CALB, Alcalase-CLEA, PPL	ChCl/Gly (1:1.5)	Aromatic aldehydes and ketones	Aldol products	First tested the lipase-catalyzed aldol reaction in DES	Gonzalez-Martinez et al. (2016)
Lipase from *Candida rugosa*	ChCl/U/Gly (1:1:1)	*p*-Nitrophenyl palmitate	*p*-Nitrophenol	Glycerol-containing DESs enhance the activity and stability more than urea-based DESs. The effects of DESs on activity and stability of lipase were partially correlated with the solvatochromic parameters. For example, the stability of lipase was correlated with hydrogen bond acidity of DESs mixtures	Kim et al. (2016)
*Thermomyces lanuginosus* lipase *Pseudozyma antarctica* lipase B	ChCl/U (1:2) ChCl/Gly (1:2)	Rapeseed oil and cooking oil	Biodiesel	Improved the additional value of cooking oil	Kleiner et al. (2016)
Lipozyme TLIM, Novozym 435	ChAc/U (2:1)	Glucose with fatty acid vinyl esters; methyl glucoside with fatty acids	Glucose-based fatty acid esters	Utilization of combination of ILs and DESs	Zhao et al. (2016)
Lipase AS	ChCl/Gly (1:2)	Aldehydes	Nitroalcohols	Addition of water could improve enzyme activity and inhibit DES-catalyzed reaction	Tian et al. (2016)
*Burkholderia cepacia* lipase	ChCl/EG (1:2)	*p*-Nitrophenyl palmitate	*p*-Nitrophenol	Significantly improved enzyme activity	Juneidi et al. (2017)
Lipase from ANL	ChCl/Gly (1:3) *Burkholderia cepacia* lipase	Dihydromyricetin	Dihydromyricetin-16-acetate	Enhancing substrate solubility	Cao et al. (2017)
iCALB	ChCl/different sugars (1:1)	Fatty acid esters	Glycolipids	Sugar can serve as HBD and substrate	Siebenhaller et al. (2017)
Lipase G	ChCl/xylitol (1:1)	Glyceryl trioleate	Epoxidized vegetable oils	DES stabilized the enzyme	Zhou et al. (2017a)
iCALB	ChCl/Gly (1:2)	Benzoic acid and glycerol	α-Monobenzoate glycerol	Water as co-solvent enzyme remained active in high concentration of DES (92%,v/v)	Guajardo et al. (2017)
PPL	ChCl/U (1:2)	Amines with aryl halides	*N*-aryl amines	DES acted as catalyst as well as solvent	Pant and Shankarling (2017)

**Fig. 4:**
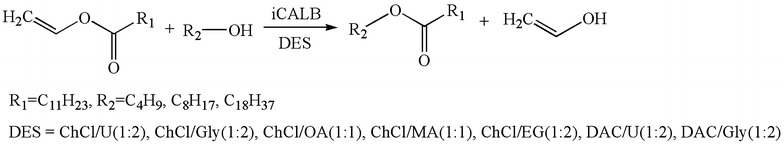
Lipase-catalyzed transesterification of vinyl ester in DESs (adapted from Durand et al. 2012)

In a following research by Durand (2012), the iCALB-catalyzed transesterification of vinyl ester was used to study the advantages and limitations of seven kinds of DESs (Fig. 4). The same competing phenomenon was observed between the DES component and the alcoholysis reaction. When the reaction occurred in ChCl:U or ChCl:Gly-containing system, alcohol substrate with different chain lengths showed no influence on the conversion and selectivity. A preliminary grinding of the immobilized enzymes could improve specific surface area in DESs, enhancing the reaction efficiency. Recently, they has intended to improve the lipase-catalyzed reaction efficiency in ChCl-based DESs (Durand et al. 2014). As mentioned earlier, albeit the liquid state of DESs at room temperature, their viscosity should be considered for the mass transfer once being used in a biocatalytic reaction. In this work, the effect of water content and urea concentration was systematically assessed. The thermodynamic activity of water (*a*_w_) was used as the study object. When the ChCl/water was lower than 1.75 in the ternary mixture, water trended to be bound to the salt and could not affect the lipase activity. In cases of higher *a*_w_, excess water molecules could affect its role as the solvent, leading to the occurrence of side reactions. When the water content in the mixture was 1 and 1.5 mol, the urea content showed no significant effect on the lipase stability. However, the denaturation effect of urea increased with the increase of urea content at higher water content.

**Fig. 5:**
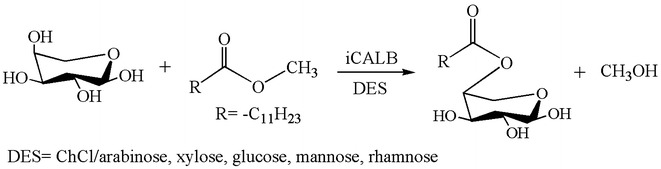
Lipase-catalyzed production of glycolipids in DESs (adapted from Siebenhaller et al. 2017)

In some cases, DES can play a bifunctional role, that is, substrate and solvent, in lipase-catalyzed reactions. Several DESs with sugars (e.g., arabinose, xylose, glucose and mannose) as HBD was used as substrate other than solvent to form glycolipid, which was detected by MS-based methods (Fig. 5) (Siebenhaller et al. 2017). This approach of using DESs exhibits an apparent advantage that high concentration of the sugar substrate can push the reaction forward in the product direction.

**Fig. 6:**
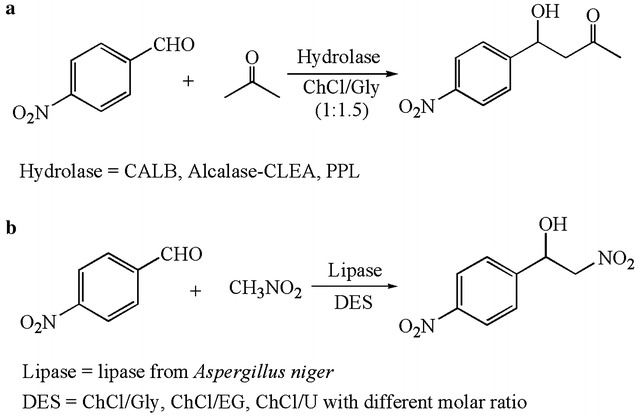
Lipase-catalyzed promiscuous reactions in DESs (adapted from (**a**) Gonzalez-Martinez et al. 2016; (**b**) Tian et al. 2016)

Lipase-catalyzed promiscuous reactions, such as carbon–carbon bond formation, Henry and Aldol reactions, have attracted some groups’ attention (Gonzalez-Martinez et al. 2016; Guan et al. 2015; Tian et al. 2016; Zhou et al. 2017b). Gonzalez-Martinez and co-workers investigated the lipase-catalyzed aldol reactions in DES-containing system in 2016 (Gonzalez-Martinez et al. 2016). A model reaction between 4-nitrobenzaldehyde and acetone was set up to examine the feasibility of DES as a reaction medium for the promiscuous aldol process (Fig. 6a). Three hydrolases, CALB, Alcalase-CLEA and PPL, were explored. Compared to the other two enzymes, PPL displayed great activity at different reaction temperatures (30–60 °C) in a system containing ChCl:Gly. The ratio of ChCl to Gly (1:1.5 or 1:2, molar ratio) posed little effect on the conversion and product composition. For this, the ratio was not probably the main aspect that affected the reaction efficiency under the optimized conditions. Different mole ratios of the HBA to HBD may change the hydrogen bonding formation, further leading to the change of the stereochemical structure of the enzyme, which affects the reaction in turn. Secondly, they tested the suitability of the ChCl:Gly-containing system by exploring different substrate groups, i.e., substituted benzaldehydes with acetone, cyclopentanone and cyclohexanone. In most cases, satisfactory results were obtained. At the same year, Tian et al. explored the Henry reaction catalyzed by lipase AS in ChCl/Gly(1:2)-containing media (Fig. 6b) (Tian et al. 2016). Water as a co-solvent was important in the reaction, not only improving enzyme activity, but also inhibiting spontaneous reaction. More recently, a chemoenzymatic epoxidation of alkenes catalyzed by CALB was reported. DES, ChCl/sorbitol, efficiently stabilized the biocatalyst, allowing the enzyme to remain active in an oxidative solvent system (Zhou et al. 2017b). The idea of trying lipase-catalyzed promiscuous reaction is of great significance to discover new functions for enzymes in DESs and synthesize many valuable chemicals.

#### Protease

**Fig. 7:**
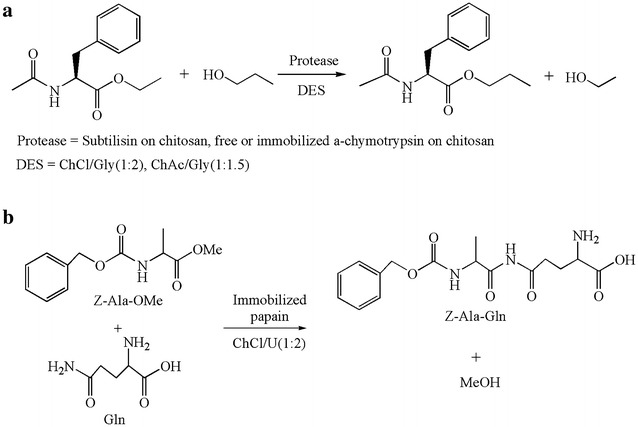
Protease-catalyzed reactions in DESs (adapted from (**a**) Zhao et al. 2011b) (**b**) Cao et al. 2015;

Proteases also find promising applications in DESs-containing systems. To explore the feasibility of DES in biocatalysis, Zhao and co-workers evaluated the protease-catalyzed transesterification activities in glycerol-based DESs based on choline salt (chloride or acetate form) (Fig. 7a) (Zhao et al. 2011b). This work indicated again the key role of water in the reaction system. While water content increased from 2% (v/v) to 4%, the activity of immobilized subtilisin exhibited a remarkable 1.8-fold increase (from 0.50 to 0.90 μmol min^−1^ g^−1^) in choline acetate/glycerol (1:1.5). This phenomenon was more obvious for immobilized α-chymotrypsin. Under reaction conditions with addition of water to the DESs solution, the biocatalytic efficiencies of these two proteases were better than those in *t*-butanol. Apparently, certain DES could activate the protease activity to some extent. This report also demonstrated that immobilization materials could function with water molecules to lower *a*_w_ to stabilize the forward reaction.

Except transesterification reaction, proteases-catalyzed peptides synthesis in DESs also proved to be successful. Different ChCl-based DESs using glycerol, urea, xylitol and isosorbide as HBDs were used as solvents for the production of peptides catalyzed by α-chymotrypsin (Maugeri and Leitner 2013). High productivities of 20 g L^−1^ h^−1^ were afforded at the optimal conditions with ChCl/glycerol and addition of 10–30% (v/v) water. The α-chymotrypsin showed good recycling ability in this DES solution system. Recently, the immobilized papain on a magnetic material was successfully used for the synthesis of *N*-(benzyloxycarbonyl)-alanyl-glutamine (Z-Ala-Gln) in ChCl/urea (1:2) as well, the yield of which reached about 71.5% (Fig. 7b) (Cao et al. 2015). In this example, more glutamine (Gln) dissolved in DES buffer remarkably improved the yield and a high Gln/Z-Ala-OMe ratio of 3–4 could effectively inhibit the side reaction to form Z-Ala-OH.

#### Epoxide hydrolase

**Fig. 8:**
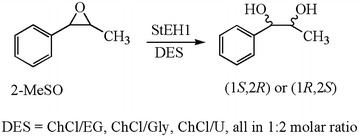
Epoxide hydrolases-catalyzed hydrolysis of 2-MeSO in DES (adapted from Lindberg et al. 2010)

Epoxide hydrolases (EH), another important hydrolase, catalyze the enantioselective hydrolysis of epoxides to the corresponding diols, which are important chiral synthetic intermediates. Up to now, there are only two examples of using DES in EH-catalyzed reactions. The first example was reported in 2008 (Gorke et al. 2008). By the addition of 25% (v/v) ChCl/Gly to the buffer, the conversion of styrene oxide catalyzed by EHAD1 increased from 4.6 to 92%. No change in the enantioselectivity was observed with the increase of conversion. The other one was carried out by Widersten et al. (Fig. 8) (Lindberg et al. 2010). Ethane diol, glycerol and urea were used as the hydrogen bond donors. The DES ChCl/Gly still exhibited a superior solvent property for this reaction than the other two DESs. Surprisingly, the regioselectivity of this hydrolysis reaction varied with the alteration of solvents. More amounts of (1*R*, 2*R*)-2-MeSO (2-methylstyrene oxide) diols were produced when the diol-and triol-containing DESs were added into the buffer as co-solvents, while the effect of glycerol on the StEH1 catalysis was the least. Many researchers considered that the decrease of enzymatic efficiency in higher ILs concentrations could be attributed to the denaturation of enzyme. However, in this work, the authors proposed that higher DES concentrations could cause the destabilization of enzyme–substrate or reaction intermediate complexes according to steady-state kinetic parameters of StEH1 at different DES concentrations. In addition, the substance concentration with the addition of DES had a 1.5-fold improvement compared to the DES-free buffer. This enhancement, to some degree, was related to the hydrogen bond in the solvent environment. The excellent dissolution-promoting ability of DES has already been used in nature product extraction (Dai et al. 2013b).

#### Other enzymes

In other examples of enzymatic reactions, many enzymes, such as haloalkane dehalogenases (Stepankova et al. 2014), benzaldehyde lyase (Maugeri and Domínguez de María 2014) and phospholipase D (Yang and Duan 2016), also showed improved reaction efficiency in the DES-containing system (Table 4). For the diglycosidase from *Acremonium* sp. DSM24697, the deglycosylation of hesperidin happened when ChCl combined with urea, glycerol and ethylene glycol was added into the buffer, whereas the enzyme activity was inhibited by DES to some extent (Weiz et al. 2016). The apparent advantage of this approach is the enhanced substrate solubility by DES. Such a property has been successfully applied in the extraction of the natural product [e.g., flavonoid (Zhao et al. 2015) and grape skin phenolics (Cvjetko Bubalo et al. 2016)]. This case is beneficial for the modification of many natural products, which have good solubility in DES.

**Table 4:** Other examples of enzymatic reactions in DESs

Catalyst	DES	Substrate	Comments	References
Haloalkane dehalogenases	ChCl/EG (1:2)	1-Iodohexane	Improved enzyme thermostability and substrate solubility	Stepankova et al. (2014)
Benzaldehyde lyase	ChCl/Gly (1:2)	Butyraldehyde; valeraldehyde; benzaldehyde; 2-furaldehyde	Improved *e.e.*	Maugeri and Domínguez de María (2014)
Phospholipase D	ChCl/EG (1:2)	Phosphatidylcholine with l-serine	>90% yield of phosphatidylserine	Yang and Duan (2016)
Diglycosidase	ChCl/Gly (1:2); ChCl/EG (1:2)	Hesperidin	Enhanced substrate solubility	Weiz et al. (2016)
Horseradish peroxidase; cytochrome c	ChCl or EAC with U, Gly and EG (1:1.5,1:2)	Guaiacol	Enhancing the functional stability of protein	Papadopoulou et al. (2016)
Chondroitinases ABCI	ChCl/Gly (1:2)	Chondroitin	Improving thermal stability remarkably	Daneshjou et al. (2017)
β-d-glucosidase	ChCl/EG (2:1)	Daidzin	Application of DES in the synthesis of bioactive compound	Cheng and Zhang (2017)
Bovine liver catalase	ChCl/EG (1:2)	Hydrogen peroxide	DES could change the Km and Kcat of enzyme in DES-containing solution. Structure test found that the 3D structure was influenced by the addition of DES	Harifi-Mood et al. (2017)

### DES as solvent for whole-cell biocatalysis

Whole-cell biocatalysis using bacteria or fungi as catalyst, e.g., *Acetobacter* sp. and recombinant *Saccharomyces cerevisiae*, has been successfully applied in some organic reactions (Gangu et al. 2009; Wachtmeister and Rother 2016; Xu et al. 2016). In comparison with isolated enzymes, whole-cell catalysts, either in the form of natural organisms or genetically recombinant strains, have a series of advantages, such as lower cost than purified enzymes (Tufvesson et al. 2011), perfect cofactor regeneration system (Hummel and Gröger 2014), applicability in untraditional media (Dennewald and Weuster-Botz 2012) and feasibility of multi-step cascade reactions in one cell (Chen et al. 2015; Peters et al. 2017).

Generally, a monophasic system forms when DES is added to aqueous buffer due to its good solubility in water. As a result, the DES-containing medium will serve as an efficient buffer system for a whole cell-catalyzed process. DES can improve, to some degree, the substrate concentration in the reaction system and further increase the reaction efficiency.

**Fig. 9:**
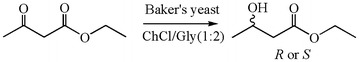
Baker’s yeast-catalyzed reduction reaction in DESs (adapted from Maugeri and de Maria 2014)

**Fig. 10:**
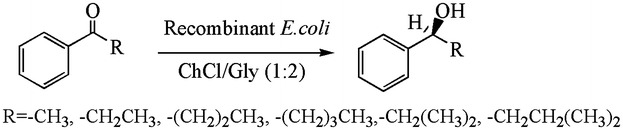
Recombinant *E. coli*-catalyzed reduction reactions in DES (adapted from Muller et al. 2015)

The first example of whole-cell biocatalysis in DES was the reduction of ethyl acetoacetate catalyzed by baker’s yeast (Fig. 9) (Table 5) (Maugeri and de Maria 2014). The yeast worked well when ChCl/Gly (1:2) was added into an aqueous buffer, and even enantioselectivity had a complete inverse when altering the proportion of DES used. The tested ChCl/Gly may go into the cell and inhibit the enantioselective enzyme activity or affect the configuration of partial enzymes. A similar enantioselectivity inversion by the addition of DES was further expanded to a series of arylpropanone substrates (Vitale et al. 2016). Both the examples used wild-type baker’s yeast as a biocatalyst. It is worth understanding how DESs affect the intracellular enzymes. Recombinant *E. coli* cells expressing oxidoreductases were also investigated for their catalytic reduction of ketones in DES-containing system (Muller et al. 2015). The cells performed surprisingly even in an 80% (v/v) DES-containing system for a broad range of aromatic substrates (Fig. 10).

**Table 5:** Examples of whole-cell biocatalytic reactions in DES

Microbial	DES	Substrate	Product	Comments	References
Baker’s yeast	ChCl/Gly (1:2)	Ethyl acetoacetate	Ethyl 3-hydroxybutyrate	Increasing the DES content switched the enantioselectivity	Maugeri and de Maria (2014)
*Acetobacter* sp. CCTCC M209061	ChCl/U (1:2)	3-Chloropropiophenone	(*S*)-3-Chloro-1-phenylpropanol	Combination of ILs and DESs in the biphasic system effectively improved the substrate concentration	Xu et al. (2015)
Recombinant *E. coli* overexpressing ADH	ChCl/Gly (1:2)	Ketones	Alcohols	The *e.e.* of many aromatic substrates increased by the addition of DES	Muller et al. (2015)
Baker’s yeast	ChCl/Gly (1:2)	Aryl-containing ketones	Aryl-containing alcohols	(*S*)-oxidoreductases of baker’s yeast was possibly inhibited by DES	Vitale et al. (2016)
Recombinant *E. coli* CCZU-T15	ChCl/Gly (1:2)	4-chloro-3-oxobutanoate	(S)-4-chloro-3-hydroxybutanoate	Addition of Tween-80 improved significantly substrate concentration from 2 to 3 M	Dai et al. (2017)
*Lysinibacillus fusiformis* CGMCC 1347	ChOAc/U (1:1) ChOAc/EG (1:1) ChCl/Lac (4:1) ChCl/Raf (11:2)	Isoeugenol	Vanillin	NADESs were first used in whole-cell biocatalysis	Yang et al. (2017)

In short, genetically modified recombinant hosts overexpressing different kinds of enzymes with different functions, especially ketoreductase (Ni and Xu 2012), open the way to the industrial production of many valuable compounds. The cost-effective, nonhazardous DESs will present greatly stimulative potential in these biocatalytic processes.

### DES as extractive agents in biotransformation

The separation of the product or unreacted substrate from a reaction should be considered in an industrial catalytic process, especially the biocatalytic reaction, regardless of the utilization of isolated or immobilized biocatalyst. DESs also show great ability to separate the desired compounds from a reaction mixture. Many products are traditionally extracted using organic liquids, such as ethyl acetate, *n*-butyl alcohol, *n*-hexane and isopropyl ether.

As mentioned earlier, DESs are often biomass derived and formed by mixing of ChCl and a hydrogen bond donor. Krystof et al. (2013) successfully separated 5-hydroxymethylfurfural (HMF) esters with HMF after the lipase-catalyzed (trans)esterification reaction, which exhibited a hydrogen donor character by using ChCl-based DES. High ester purity (>99%) and efficiency (up to >90% HMF ester recovery) were observed. This finding paved a new path for the HMF chemistry. Inspired by that, ChCl-based DES was also utilized by Qin et al. to separate HMF and 2,5-diformylfuran (DFF) from the reaction mixtures, and the purity of the latter reached up to 97% (Qin et al. 2015). The two examples discussed above to some extent were based on the characteristics of the hydrogen bond donor of the separated chemical compounds. Maybe, the formation of a new hydrogen bond existed between the DES and target compounds.

Hydrophobic DES containing quaternary ammonium salt/decanoic acid mixture could extract volatile carboxylic acids from aqueous solution (van Osch et al. 2015). The DESs extracted acetic acid, propionic acid and butyric acid more efficiently than a traditional solvent like trioctylamine. For example, DecA:N_8881_-Cl extracted twice as much acetic acid as trioctylamine (38.0% vs 18.6%). DecA:N_7777_-Cl extracted 91.5% of the butyric acid. This kind of DES costs less than some ammonium and phosphonium ionic liquids for extracting butyric acid (Blahušiak et al. 2013; Marták and Schlosser 2016). However, another challenge about the separation of acid from DES is put forward subsequently. The traditional distillation method could facilitate the process owing to the extremely low volatility and high thermal stability of DESs (Wu et al. 2015).

### DESs function in biomass pretreatment

DES pretreatment of biomass is a good alternative of IL-based processes. Currently, most attention has been focused on the use of ILs, especially imidazole- and choline-based ILs (Sheldon 2016), for preprocessing cellulose-based biomass to improve the following enzymatic hydrolysis process indirectly. DESs are able to dissolve biopolymers like polysaccharides by breaking the supermolecular structure formed by intermolecular hydrogen bonds (Ren et al. 2016). For example, ChCl/imidazole can be used to pretreat corncob at a relatively low temperature of 80 °C and the final glucose yield reached 92.3% after the enzymatic hydrolysis (Procentese et al. 2015). Due to the diversity of HBDs, researchers have more choice of using different HBDs in conjunction with ChCl for pretreatment; thus, different results are obtained normally, even for the same biomass like corncob (Zhang et al. 2016). Considering the relative cheapness of ILs, it is useful to develop more cellulase-compatible DESs, making it possible to omit the separation of DESs from the system.

## Influence of DESs on biocatalysis

As shown in the last chapter, many positive examples show the great promising future of DESs in biocatalysis. It is of interest to elucidate under what circumstances and how the biocatalysts retain their biological function and stability in DESs.

### Effect of the characteristics of DESs on the biocatalytic process

The tunability of DES contributes to much more possibility of designing vast kinds of DES, thus bringing various effect on a biocatalytic reaction system. The selection of HBAs and HBDs make a big difference in the whole process. For instance, ChCl/Gly is usually used as a good solvent/co-solvent for enzymatic reaction, while the performance of ChCl/MA is not satisfactory (Durand et al. 2012). Different HBDs also form DESs with different viscosities (Zhang et al. 2012), which affects the mass transfer of all the reactants (substrate, product, catalyst, etc.) and further changes the reaction rate. At this point, the hydrogen bond is another concern posed by various HBDs. There is a difference of just one hydroxyl group between Gly and EG; however, the resulting DESs showed remarkable difference on the lipase-catalyzed reaction (Durand et al. 2012).

Hydrogen bonding of DESs is the main power which makes them distinctive with their individual components. This special force can activate the enzyme by increasing the enzyme affinity with the substrate (Juneidi et al. 2017). It is also necessary to consider the ratio of HBA/HBD, which might affect the solvatochromic parameters and the hydrogen bond formation between the reaction mixtures (Kim et al. 2016). A molecular dynamics simulation of lipase in ChCl/U firstly proved that the hydrogen bond between DES components could hinder the attack of U to the enzyme function domains, thus resulting in a stabilized enzyme (Monhemi et al. 2014).

In addition, DES concentration and water content are two interacting factors with significant influence on the reaction efficiency. A high amount of water not only decreased the viscosity of the DES solution, but also enabled the contact between the enzyme molecule and substrate (Guajardo et al. 2017). But too much water might have a destructive effect on the hydrogen bond network of DES and weaken the benefit of DES.

### Influence of DESs on biocatalyst structure and activity

Firstly, DESs can change the secondary structure of enzymes. Take the horseradish peroxidase (HRP) for example (Wu et al. 2014): higher HBD molar ratio (e.g., from 2:1 to 1:2) in ChCl-based DES increased the α-helix content in the HRP. More α-helix and less β-sheet in the structure were beneficial for their activity and stability. The improvement in activity may result from the looser tertiary structure of HRP. A recent report confirmed that the ChCl component had a disruptive influence on the α-helix, which would cause a decrease of the enzyme activity. However, the glycerol component could markedly increase the α-helix content and decrease the β-sheet content, leading to a more stable enzyme. Thus, a trade-off effect between HBA and HBD, either activation or deactivation, can be observed for a certain DES.

Secondly, DESs can affect the 3-D structure of enzymes in an aqueous environment. The functional configuration of enzymes is essentially related to the folding and unfolding degree. Several factors including hydrophobicity, solvent polarity and hydrogen bond characteristics of ILs can affect separately or together the protein stability and activity (Weingartner et al. 2012). Similarly, the concentration of DESs in an aqueous solution had significant effect on the unfolding and refolding process of the enzyme, which could be determined by spectroscopy technologies such as intrinsic fluorescence and CD spectroscopy (Esquembre et al. 2013). The two methods can effectively estimate the folded degree of proteins compared to the native-like secondary and tertiary structures. A redshift of the fluorescence emission spectra of tryptophan residues in lysozyme was observed when tryptophan side chain was exposed to a polar surrounding of water molecules. In neat ChCl/U and ChCl/Gly solution, the emission maximum of lysozyme shifted from 335 nm (observed in buffer) to 332 and 329 nm, respectively (Esquembre et al. 2013). However, higher concentration of DESs can cause the irreversible unfolding of lysozyme at high temperatures. Meanwhile, the protein accumulation in low content of DESs buffer at room temperature was interestingly reversible.

In whole-cell biocatalysis, DESs increased the permeability of cells, lowering resistance to mass transfer resistance, thereby improving the reaction efficiency (Xu et al. 2016). ILs similarly can improve the permeability of cells (Xiao et al. 2012). In other cases, DESs can enhance the affordable substrate concentration for a cell catalyst, thereby improving the catalytic rate and yield of the product (Xu et al. 2016). The worst situation is that DESs possibly react with the cell membrane and induce cell apoptosis, finally leading to cell death.

## Conclusion and perspective

Deep eutectic solvents are easier to prepare with different kinds of biocompatible and naturally occurring constituents. Varying the component and ratio yields DESs with different T_*f*_, polarity, density and viscosity. Some DESs show toxic profile toward laboratory organisms, which depends on the used components, test conditions and organisms. However, most DESs can be considered as “readily biodegradable” solvents. DESs can work as solvents, co-solvents or extracting solvents in a specific biocatalytic reaction. Remarkably, as solvents, DESs (e.g., Gly-based DESs) can activate and stabilize the enzyme, thus achieving a high reaction efficiency. Multiple hydrolases (lipase, protease and epoxide hydrolase) and other enzymes exhibit great catalytic performance in these solvents, which displays their vast potential to replace ILs and organic solvents in biocatalytic reactions. An in-depth understanding of how DESs activate and stabilize enzymes will promote the application of DESs in biocatalysis to step off laboratory scale.

However, DESs in biocatalysis needs researchers to fulfill their full physical–chemical characterization and the important toxicity data. The plausible relationship between the structure and function of enzymes and DESs will be an interesting field to explore. Additionally, the downstream separation of target product from DESs is also a big challenge. More utilization of environmental-friendly and economical solvents in biocatalysis will embody the greenness and sustainability of green chemistry.

## Abbreviations

ChCl: choline chloride
MTPB: methyltriphenylphosphonium bromide
DAC: *N,N*-diethyl ethanol ammonium chloride
Gly: glycerol
EG: ethylene glycol
DEG: diethylene glycol
TEG: triethylene glycol
U: urea
A: acetamide
OA: oxalic acid
MA: malonic acid
PTSA: para-toluene sulfonic acid
Glc: glucose
Lac: lactose
Raf: raffinose
Fru: fructose
Suc: sucrose
ZnN: zinc nitrate hexahydrate
Choline and geranate: choline bicarbonate/geranic acid
PC3: human prostate cancer cell line
A375: human malignant melanoma cell line
HepG2: human liver hepatocellular cell line
HT29: human colon adenocarcinoma cell line
MCF-7: human breast cancer cell line
OKF6: human oral keratinocyte cell line
H413: carcinoma-derived human oral keratinocyte cells
HelaS3: human cervical cancer cell line
CaOV3: human ovarian cancer cell line
B16F10: mouse skin cancer cel line
AGS: human gastric cancer cell line
WRL-68: human hepatocyte cell line
CALA * Candida **antarctica* lipase A iCALB: *Candida antarctica* lipase B immobilized on acrylic resin
CALB: *Candida antarctica* lipase B
CRL: *Candida rugosa* lipase
PCL: *Pseudomonas cepacia* lipase
PPL: *Porcine pancreas* lipase
Alcalase-CLEA: protease from *Bacillus licheniformis*
Lipase AS: lipase from *Aspergillus niger*
StEH1: potato epoxide hydrolase
EHAD1: epoxide hydrolase from *Agrobacterium radiobacter* AD1
BMIM[Tf_2_N]: 1-butyl-3-methylimidazolium bis(trifluoromethanesulfonyl) imide
[BMPyr][NTf2]: 1-butyl-1-methylpyrrolidinium Bis(trifluoromethanesulfonyl)imide
[Bmim]HSO4: 1-butyl-3-methylimidazolium hydrosulfate
